# Entry Aggregation and Early Match Using Hidden Markov Model of Flow Table in SDN

**DOI:** 10.3390/s19102341

**Published:** 2019-05-21

**Authors:** Cheng Wang, Hee Yong Youn

**Affiliations:** 1 Electrical and Computer Engineering, Sungkyunkwan University, Suwon 16419, Korea; lighting91@skku.edu; 2 College of Software, Sungkyunkwan University, Suwon 16419, Korea

**Keywords:** SDN, entry aggregation, Quine–McCluskey Algorithm, match frequency and probability, Hidden Markov Model

## Abstract

The usage of multiple flow tables (MFT) has significantly extended the flexibility and applicability of software-defined networking (SDN). However, the size of MFT is usually limited due to the use of expensive ternary content addressable memory (TCAM). Moreover, the pipeline mechanism of MFT causes long flow processing time. In this paper a novel approach called Agg-ExTable is proposed to efficiently manage the MFT. Here the flow entries in MFT are periodically aggregated by applying pruning and the Quine–Mccluskey algorithm. Utilizing the memory space saved by the aggregation, a front-end ExTable is constructed, keeping popular flow entries for early match. Popular entries are decided by the Hidden Markov model based on the match frequency and match probability. Computer simulation reveals that the proposed scheme is able to save about 45% of space of MFT, and efficiently decrease the flow processing time compared to the existing schemes.

## 1. Introduction

In software-defined networking (SDN) [1,2] efficient network management is achieved by separating the control plane from the data plane, and providing programmable interfaces to the application layer to flexibly enable the customization of the network management functions. The traditional legacy network services often require various processing of data packets such as routing, monitoring, access control, and load balancing. If all the services are provided in a single network infrastructure, the development of the network will be very complicated and adapting to a variety of functional requirements will be difficult. In this regard, SDN provides a new direction of networking services and drastically changes the traditional network with distinctive features including the separation of controller and forwarding unit, virtualization, programmable service, dynamic reconfiguration, and centralized management [3].

SDN is an innovative movement in the field of computer networks. It stems from a series of projects on networks such as active networks, software switches, 4D (Decision, Dissemination, Discovery, and Data) networks, and traditional telephone networks [4,5]. The network infrastructure has been evolving from the approach of vertical integration to horizontal one to be more intelligent. Intelligent control of data forwarding units (switches, routers, etc.) has been unified into the control plane [2]. With SDN the network devices of the data plane only forward the data, while they are directly managed by the control plane. As a result, the control plane can get the global information of the network such as connection topology, link state, etc., and thus proper decisions on forwarding the flows can be made. The network is programmable through a unified interface provided to control the behavior of the whole network [6]. The programmable interface greatly improves the flexibility of network management and data forwarding, and enables the network to dynamically change the path of the flow. In SDN, the physical characteristics of the networking devices are concealed so that the control plane can provide a unified management and services for high level applications [7]. At the same time, the data forwarding devices are abstracted from the control layer to ensure the mobility, and reduce future investment of the devices.

Among the main issues with SDN such as switch designs [8,9], distributed controller platforms [10,11], resilient communication [12,13], and security [14,15], flow table management is one of the primary tasks directly influencing the performance. From OpenFlow 1.3 [16,17], there were several improvements including multiple flow tables (MFT). In SDN, the OpenFlow switches just forward the flows through the MFT which are composed of data packets, and the operation is managed by OpenFlow controllers. Even though MFT plays an important role in SDN, two challenging issues exist which are memory overhead and flow forwarding delay. The memory overhead is high due to the need of ternary content addressable memory (TCAM) and fast forwarding [18]. If the number of entries in the table is limited too much for reducing the overhead, the flow match rate will be very low. Meanwhile, the pipeline mechanism employed with MFT causes forwarding delay as each flow has to go through the flow table one after another, and the action corresponding to the matched entry is executed at the end of the pipeline. There exist various studies dealing with such issues of MFT. For example, the Flow Table Reduction Scheme [18] (FTRS) aggregates the flow entries of the same action and destination addresses. In [19], the Multi-Stage OF (MsOF) switch model is proposed to save memory space and reduce forwarding time by deploying the tables each requiring less memory space. However, the FTRS works well only in local area network (LAN) environment and the implementation of MsOF is relatively complicated.

In this paper we advocate a novel approach called Agg-ExTable which efficiently reduces the memory overhead and flow forwarding time of MFT. Here pruning and the Quine–McCluskey (QM) algorithm are periodically applied to aggregate the flow entries in each flow table, and a table called ExTable is put in front of the pipeline which contains the flow entries of high expected match probability. Its flow entry is dynamically determined using a Hidden Markov model constructed based on the number of transitions and match probability of the entries. The QM algorithm allows an efficient usage of TCAM by reducing the size and power consumption, while ExTable allows quick match and execution of the incoming flows. Computer simulation reveals that the proposed scheme substantially reduces the memory size and flow processing time compared to the TCAM-based size reduction scheme [20] and the scheme merging and migrating the flow entries based on directed acyclic graph (DAG) [21]. The improvement gets more significant as the flow arrival rate rises. The main contributions of the paper are summarized as follows:

- The flow entry aggregation problem is simplified by transforming it to a logic minimization problem, which is effectively solved by the QM algorithm.
- The ExTable scheme substantially reduces the flow processing time by placing a table containing the entries of high match probability up front.
- The match frequency and match probability of the entries are handled with the Hidden Markov model to decide the likelihood of the match.

The remainder of the paper is organized as follows. Section 2 provides an overview of TCAM used for the implementation of MFT, Hidden Markov model, and MFT-based switching. In Section 3 the proposed Agg-ExTable approach is presented, along with the analytical model of the flow processing time based on queueing theory. Section 4 is for the performance evaluation of the proposed schemes. Finally, Section 5 concludes the paper and outlines the future research direction.

## 2. Related Work

### 2.1. TCAM

TCAM [20,22] is usually employed to speed up the table look up operation. However, the size is always limited due to high cost. TCAM allows a third matching state of ‘X’ or ‘don’t care’ for one or more bits in the stored entry, allowing the flexibility in the search. For example, a TCAM may have an entry of ‘101XX’ which will match to ‘10100’, ‘10101’, ‘10110’, or ‘10111’. The additional state is typically implemented by adding a mask bit to the corresponding memory cell. If a bit of the mask is ‘0’, the bit of corresponding entry is ‘don’t care’. In Table 1, for example, if the first five bits of a flow are ‘10011’ or ‘10111’, it matches *E*_1_.

Another important feature of TCAM is that ‘0’ and ‘1’ in the mask is not required to be continuous. For *E*_1_ in Table 1, the third bit of the entry is ‘don’t care’, and thus the flow whose first three bits are ‘100’ or ‘101’ matches this entry. The proposed aggregation method takes advantage of this feature of TCAM.

In reference [20], two techniques, pruning and ESPRESSO-II based mask extension, are proposed to compact traditional routing table stored in TCAM. They allow a smaller TCAM reducing the size and power consumption.

### 2.2. Quine–McCluskey (QM) Algorithm

The Quine–McCluskey (QM) algorithm [23,24,25] was developed for simplifying logical functions. The QM algorithm is effective for implementation since it has tabular form, and it also provides a deterministic method checking if the logical function is minimal. There exist mainly four definitions in using the QM algorithm:

- Minterm: an expression in which all variables of the logical function appears once.
- Implicant: an aggregation of minterms in the logical function.
- Prime implicant: an implicant that cannot be covered by a more simplified implicant.
- Essential prime implicant: prime implicants that cover an output of the logical function for which no combination of other prime implicants is able to cover.

The procedure of the QM algorithm consists of two steps:

- Step 1: Find prime implicants from the minterm table.
- Step 2: Find the essential prime implicants and other necessary prime implicants to cover the logical function.

In the proposed scheme the QM algorithm is employed for mask extension which is transformed to be a logic minimization problem.

### 2.3. Hidden Markov Model (HMM)

HMM [26,27,28,29] is used in various fields such as language recognition, reinforcement learning, and bioinformatics. An HMM is a finite discrete time Markov model in which the system is assumed to be a Markov process with hidden state. It can be defined as a triple *λ* = (*π*,*A*,*B*), where *π* is the initial probability distribution, *A* is transition probability matrix, and *B* is a sequence of observation likelihoods (emission probabilities). Specifically, an HMM is defined by the following components [30]:

- *Q* = {*q*_1_, *q*_2_, …, *q_N_*} is a set of states where *N* is the number of hidden states, and *q_t_* is the hidden state at time *t*.
- *O* = {*o*_1_, *o*_2_, …, *o_T_*} is a set of observations where *T* is the number of observations. *o_t_* is the observable state at time *t*. Each observation is drawn from a vocabulary *V* = {*v*_1_, *v*_2_, …, *v_M_*}, where *M* is the number of observation values.
- *A* = {*a*_11_, *a*_12_, …, *a_N_*_1_, …, *a_NN_*} is an *N* × *N* transition probability matrix. *a_ij_* (1 ≤ *i*, *j* ≤ *N*) represents the probability of changing from state_*i* to state_*j*. Here,
  (1) $$a_{ij}=P(q_{t+1}=s_{j}|q_{t}=s_{i}),$$
  while,
  (2) $$a_{ij}\geq 0,\;\forall i,j,$$
  (3) $$\sum_{j=1}^{N}a_{ij}=1,\;\forall i.$$
- *B* = {*b_j_*(*k*)}(1 ≤ *j* ≤ *N*, 1 ≤ *k* ≤ *M*) is an *N* × *M* emission probability matrix. *b_j_*(*k*) represents the probability of an observation *o_t_* being generated from state_*j*. Here,
  (4) $$b_{j}\left(k\right)=P\left(o_{t}=v_{k}|q_{t}=s_{j}\right),$$
  while,
  (5) $$\sum_{k=1}^{M}b_{j}\left(k\right)=1,\;\forall j.$$
- *π* = {*π*_1_, *π*_2_, …, *π_N_*} is the initial probability distribution over hidden states. *π_i_* is the probability that the Markov chain will start in state_*i*. Here,
  (6) $$\pi_{i}=P\left(q_{1}=s_{i}\right),$$
  while,
  (7) $$\sum_{i=1}^{N}\pi_{i}=1,\;\forall i.$$

**Figure 1:**
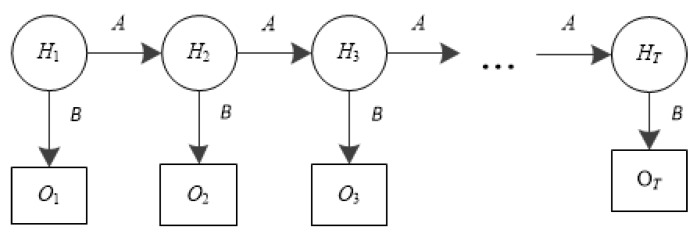
The state diagram of the Hidden Markov model.

A generic HMM is illustrated in Figure 1, where *H_i_*(*i* = 1, 2, 3, …, *T*) is the set of hidden states and *O_i_*(*i* = 1, 2, 3, …, *T*) is the set of observations. The Markov process, located above the observable states, is determined by the current state and *A*. Only *O_i_* is able to be observed, which is determined by the hidden states of the Markov process and *B*.

### 2.4. MFT

**Figure 2:**
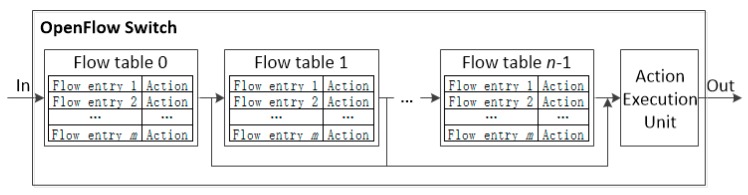
The pipeline processing with MFT.

The pipeline processing of OpenFlow [16,17] is depicted in Figure 2. An OpenFlow switch is required to have one or more flow tables, while a single flow table is used only for relatively simple network.

The flow tables of an OpenFlow switch are sequentially numbered, starting from 0. The pipeline processing always starts from the first flow table where the packet is matched against its flow entries. Other flow tables may be used depending on the outcome of the match. The pipeline processing occurs only in the forward direction. If a flow entry matches, the instruction set included in that entry is executed at the Action Execution Unit (AEU) located at the end of the pipeline.

The existing flow entry aggregation techniques can be classified into two types according to the location of aggregation, in packet classifier or in OpenFlow switch flow table. The TCAM Razor [31] is a systematic flow aggregation algorithm which uses the decision diagram to minimize the TCAM rules required for packet classification. However, the execution time grows rapidly when the number of flow entries increases. The Fast Flow Table Aggregation [32] (FFTA) and FTRS also support entry aggregation in SDN. FFTA is an offline aggregation scheme based on bit weaving, which applies ORTC (Optimal Routing Table Constructor) after cutting the entries using a binary search tree. The FTRS aggregates the flow entries according to the destination IP address, instead of the match field. It achieves a good compression ratio with different topologies. However, FFTA causes coarse traffic statistics due to the mixture of all the entries, while FRTS has a high probability of flow table overflow.

The schemes of [19,33] aim to implement effective forwarding with MFT. In reference [21], an algorithm called migrating flow rules (MILE) was proposed which merges and migrates the flow entries to reduce the number of flow table lookup operations by employing directed acyclic graph (DAG). The dependencies of the flow entries are handled using DAG, where interdependent entries are grouped and migrated as a whole. Using the 2Q LRU replacement algorithm, the recently accessed entries are replaced at Flow Table_0 to be matched early. With the Multi-Stage OF (MsOF) [19] switch model, more tables each requiring less memory space are deployed. Here a processor is implemented in each flow table, and multiple pipeline operations occur in parallel so that several flows can be matched at the same time. The spatial and temporal complexity were examined using queuing theory. The implementation of MsOF is relatively complicated and needs large networking resources.

In order to improve the efficiency of the MFT, both the TCAM implementation and flow match probability are focused in this paper. The proposed scheme aggregate the entries using the pruning and QM algorithm to minimize the space of TCAM, while the saved memory space is used to store popular entries. With the HMM constructed based on the match frequency and match probability of the entries, the presumably popular flow entries are selected and put in the ExTable located in front of the flow table. The pruning and QM algorithm aim to maximize the efficiency of the TCAM by reducing the size and power consumption. Maintaining high match rate with ExTable substantially decreases the overall flow processing time, via early match and execution of the incoming flows. The proposed scheme is presented in the next section.

## 3. The Proposed Scheme

### 3.1. The Structure

**Figure 3:**
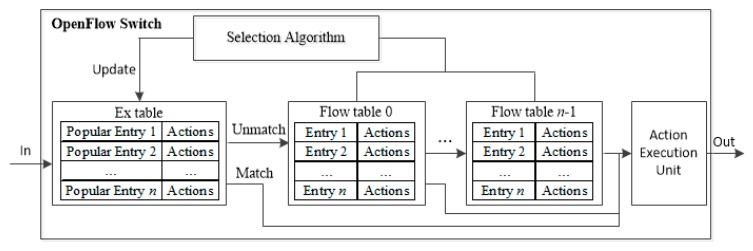
The structure of the proposed scheme.

The proposed Agg-ExTable scheme allows entry aggregation and fast pipeline operation using ExTable holding popular flows. It works in two phases. In Phase 1, the proposed entry aggregation algorithm is executed periodically to reduce the size of TCAM. Then in Phase 2, the saved memory space is utilized to set up ExTable in front of the pipeline, keeping popular entries. Here the HHM is used to decide the popularity of the flow entry. The structure of the proposed MFT pipeline of an OF switch is illustrated in Figure 3. Observe that there exist two paths; express path for popular flow and regular path for nonpopular flow. When a flow arrives at a switch, it is first parsed for the matching with the ExTable. Upon a match, the actions of the flow entry are sent to AEU. Otherwise, the match operation is continued with the other flow tables.

### 3.2. Aggregation of Entry

The number of possible flow paths in a flow table is typically small because only a limited number of interface cards can fit into the switch chassis. In contrast, the number of forwarding entries is quite large, in the range of several thousands. Considering this disparity, a scheme reducing the size of flow table is developed which involves two techniques presented below.

#### 3.2.1. Pruning of Redundant Entries

Pruning is a technique eliminating some redundant entries [20]. To facilitate the discussion, some terms are defined as follows. Notice that the match fields of a flow entry may have different lengths.

- Assume that *entry*_*P* is the parent of *entry*_*Q*, *Lp* is the length of *entry*_*P*, and *P*(*i*) is the *i*th bit of *entry*_*P*. Then the following three conditions hold: (a) *L_P_* < *L_Q_*; (b) For all *i*(1 < *i* < *L_P_*), *P*(*i*) = *Q*(*i*); (c) There is no *Q*’ such that *L_P_* < *L_Q’_* < *L_Q_*; and *Q*’(*i*) = *Q*(*i*) for all *i*(1 < *i* < *L_P_*).
- *Entry*_*P* is identical to *entry*_*Q* if same actions are executed for the matched packet.

If *P* is identical to *Q*, *Q* is a redundant flow entry. Assume that *Q* matches a flow. Then the flow will match *P* as well by the definition. If *Q* is removed from the flow table, *P* becomes the matched entry. As *P* and *Q* have the same actions, removing *Q* makes no difference. Note that the technique is general enough that it can be used with any entry lookup algorithm regardless of the type of the flow table.

#### 3.2.2. QM-Based Mask Extension

The second technique exploits the flexibility offered by the TCAM hardware. TCAM allows arbitrary mask, in other words, the bits of 1s or 0s do not require to be continuous. 

Table 2 shows an example of mask extension. *E*_1_ and *E*_2_ both have the same action of ‘Forward to 1’. It is possible to combine *E*_1_ and *E*_2_ into one single entry with the prefix of 1100 and mask of 1101. The 0 at bit 3 in the mask allows combining *E*_1_ and *E*_2_ into a same entry. The aggregated version of the original flow table with the mask extension technique is shown in the right-hand side. The table size has been reduced to 3 from 5.

Note that the mask extension is equivalent to the logic minimization problem [20]. The problem is that ‘given a set of entries with the same action, find a set of minimal covers.’ Such logic minimization problem [33] is a non-deterministic polynomial (NP) complete problem, and there exist mainly three kinds of methods used for its solution.

- Karnaugh mapping [34]: It is simple but when the number of variables is larger than six, it becomes very complex.
- Quine–McCluskey (QM) algorithm [23,24,25]: It is functionally identical to Karnaugh mapping, but the tabular form makes it more efficient to be used with a computer algorithm, supporting any number of variables. It also provides a deterministic method checking if the logical function is minimal.
- Espresso logic minimizer [35,36]: It can produce a solution fast but cannot guarantee optimal result.

Here the QM algorithm is employed for mask extension. Algorithm 1 shows the proposed entry aggregation scheme with the QM algorithm-based mask extension. Here, *E*(*l*,*a*) is the set of original entries having the same length of *l* and action of *a*. *A*(*l*,*a*) is the result of QM algorithm.

**Algorithm 1.** Entry aggregation with mask extension  //*n* is the number of original entries  //*m* is the number of entries having the same length and action  **1: Begin**  **2: Input**
*entry*[*i*]  **3: for**
*i* from 0 to *n*  **4:** **if** have same *entry*[*i*].*l* and *entry*[*i*].*a*  **5:**  move to *E*(*l*,*a*)  **6: End for**  **7: for**
*i* from 0 to *m*  **8:** *A*(*l*,*a*) = **QMminimize**(*E*(*l*,*a*))  **9: end for****10:** remove *E*(*l*,*a*)**11:** install *A*(*l*,*a*) 
**12: end**


An example of the proposed mask extension scheme is shown below. In Table 3, there are 11 entries with different actions. After selecting the entries having the same action, the entry aggregation problem is simplified into the following minimization function:

(8) F(A,B,C,D)=∑E(0,1,3,4,5,6,7,8,9).

Step 1: find prime implicants (*P_i_*). Here all minterms are placed in the minterm table as shown in Table 4, and Stage I is to combine the minterms. If two terms vary by only a single bit, that bit is replaced with a dash (-). Stage II is the result of Stage I, and Stage III is the result of combining the minterms in Stage II. ‘/’ indicates if the entry is combined in the next stage.

According to Table 4, all the prime implicants are shown as follows:

(9) $$P_{1}=\sum E\left(0,3,9,5\right)=BD,\;P_{2}=\sum E\left(8,5\right)=ACD,\;P_{3}=\sum E\left(6,8\right)=AB^{\prime }C,\;P_{4}=\sum E\left(7,6\right)=AB^{\prime }D^{\prime },\;P_{5}=\sum E\left(4,0\right)=A^{\prime }BC^{\prime },\;P_{6}=\sum E\left(1,7\right)=B^{\prime }C^{\prime }D^{\prime },\;P_{7}=\sum E\left(1,4\right)=A’C’D’.$$

Step 2: find essential prime implicants (*P_e_*).

From Table 4, none of the minterms can be combined any further. At this point, the table of essential prime implicant is constructed as in Table 5.

In order to find the essential prime implicants, each column needs to be checked whether there exists only one ‘*’. If a column has only one ‘*’, the minterm can be covered by only one prime implicant. Then this prime implicant is essential. According to Table 5, *P*_1_ is the only essential prime implicant. 

As *P*_2_ can be covered by *P*_1_ and *P*_3_, same as *P*_3_, *P*_4_, *P*_5_, *P*_6_, and *P*_7_, they are not essential. In this example, the essential prime implicants cannot handle all the minterms (only *E*_0_, *E*_3_, *E*_5_, and *E*_9_ are covered). Therefore, other prime implicants are combined with *P*_1_ to get the final result as:

(10) $$F\left(A,B,C,D\right)=P_{1}+P_{2}+P_{4}+P_{5}=BD+ACD+AB^{\prime }D^{\prime }+A^{\prime }BC^{\prime }.$$

At last the final Table 6 is obtained as follows:

The number of entries is aggregated from 11 to 6, and the compression ratio is 6/11 = 0.545.

### 3.3. Hidden Markov Model-Based Prediction

The goal of the proposed scheme based on HMM is to dynamically predict the popularity of the flow entries as accurately as possible and update the ExTable accordingly. After the flow entries are aggregated, the popularity of the flow entries are estimated periodically and the entries deemed to be popular are moved to ExTable. Note that the size of ExTable, *n_p_*, is smaller than (1 − *C*)∙*N_MFT_* where *C* and *N_MFT_* are the TCAM compression ratio and the size of entire MFT, respectively. 

The match frequency of an entry indicates the popularity. For this, a counter, *M*, is associated to each flow entry, which is activated when the entry is installed in the flow table. *M* is the number of matches before the prediction occurs. Note that *M* may not be the only indicator of the popularity, and thus HMM is employed to estimate the probability of the flow entries to be matched in the near future.

The interarrival time of the flow is assumed to follow exponential distribution, and therefore the number of arrivals can be modeled using Poisson distribution. The probability of a flow arriving in a given interval of time of Δ*t* is predicted as follows. Assume that flow arrival occurs at any time. The probability of *k* arrivals in Δ*t* is given by:

(11) $$P\left(k,\;\Delta t\right)=\frac{e^{-\lambda \Delta t}{\left(\lambda \Delta t\right)}^{k}}{k!}.$$

The probability of at least one arrival in the interval Δ*t* is given as:

(12) $$P\left(k\geq 1,\;\Delta t\right)=1-e^{-\lambda \Delta t}.$$

The probability of a flow arriving in the next interval is computed as the mean value of the entire period from the beginning. It is:

(13) $$P\left(k=1,\;\Delta t\right)=e^{-\lambda \Delta t}\cdot \left(\lambda \Delta t\right).$$

**Figure 4:**
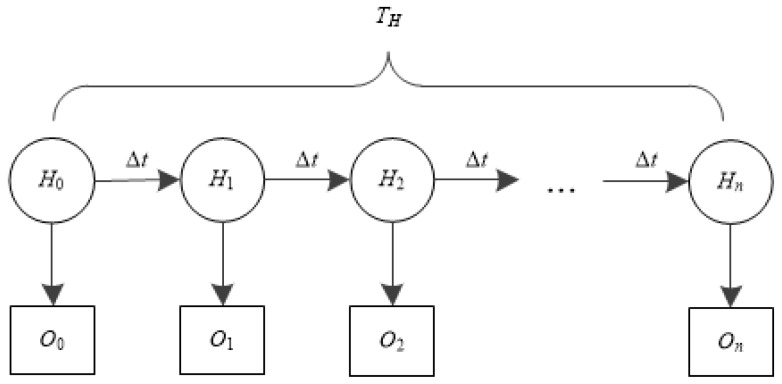
The state diagram of the proposed HMM.

HMM is effective to predict the probability of an observed sequence with the given triple *λ* = (*π*,*A*,*B*). Let *H* = {*H*_0_, *H*_1_, …, *H*_n_} be a set of hidden states, where *H_i_* is defined as the number of time segments (Δ*t* seconds per segment) a flow entry has not been matched from the initial state. For example, if there was no match for last 3Δ*t* seconds, *H*_3_ = 3Δ*t*. If there was a match, *H*_3_ = 0. Note that the hard timeout period (*T_H_*) is preset, and an entry is forced to be evicted if no match occurs during *T_H_*. Therefore, there will be *n* (=*T_H_*/Δ_t_) segments before an entry is finally evicted, and *H_n_* is the last state. Since *O* = {*O*_0_, *O*_1_, …, *O_n_*} is a set of observable states of any entry, *O_i_* indicates if the entry is matched in *i*th segment. It has two values; ‘1’ for a successful match, ‘0’ no match. Figure 4 shows the structure of the HMM of the proposed scheme.

With the HMM the probability of an observed sequence is found with the given parameters, *A*, *B*, and *π*. For a flow entry, there exist *N* (=*n* + 1) hidden states in its life time. Assume that there exist *m* time segments before the prediction occurs. Then the (*n* − *m*) × (*n* − *m*) hidden state transition probability matrix, *A*, and the (*n* − *m*) × 2 emission probability matrix, *B*, are obtained as follows:

(14) $$A=\begin{array}{c} \begin{array}{c} \left[\begin{array}{cc} \begin{array}{cc} 1-e^{-\lambda \Delta t} & e^{-\lambda \Delta t}\\ \begin{array}{c} 1-e^{-\lambda \Delta t}\\ \begin{array}{c} \vdots \\ \begin{array}{c} 1-e^{-\lambda \Delta t} \end{array} \end{array} \end{array} & \begin{array}{c} 0\\ \begin{array}{c} \vdots \\ \begin{array}{c} 0 \end{array} \end{array} \end{array} \end{array} & \begin{array}{cc} \begin{array}{c} \begin{array}{c} \begin{array}{c} 0\\ e^{-\lambda \Delta t} \end{array}\\ \vdots \end{array}\\ 0 \end{array} & \begin{array}{cc} \begin{array}{c} \begin{array}{c} \begin{array}{c} \ldots \end{array}\\ \vdots \end{array}\\ \vdots \\ \ldots \end{array} & \begin{array}{c} \begin{array}{c} \begin{array}{c} \begin{array}{c} 0\\ 0 \end{array}\\ \vdots \end{array}\\ e^{-\lambda \Delta t} \end{array} \end{array} \end{array} \end{array} \end{array}\right] \end{array} \end{array}\;,$$

(15) $$B=\begin{array}{c} \begin{array}{c} \left[\begin{array}{cc} 0 & 1\\ 1 & 0\\ \begin{array}{c} \vdots \\ 1 \end{array} & \begin{array}{c} \vdots \\ 0 \end{array} \end{array}\right] \end{array} \end{array}.$$

In order to compute the likelihood probability, *P*(*O*|*λ*) of *O* = {*O*_0_, *O*_1_, …, *O*_(*n*−*m*)_}, the forward algorithm is adopted. Note that the probability of the observation sequence is obtained in which the value of *O_i_* is 1 and the predicted last observable state is *O*_(*n*−*m*)_. Then *P*(*O*|*λ*) is calculated as follows.

1. Initialization. Each cell of the forward algorithm, *α_t_*(*j*), represents the probability of hidden state *H_j_* after checking the first *t* observations with the given *λ*. It expresses the probability as:
  (16) $$\alpha_{t}\left(j\right)=P(o_{0},o_{1},\cdots ,o_{t},H_{t}=q_{j}|\lambda ).$$
  Here *H_t_* = *q_j_* denotes that the *t*th hidden state in the sequence is *q_j_*. Then the initial probability is calculated as follows:
  (17) $$\alpha_{0}\left(j\right)=\pi_{j}b_{j}\left(o_{0}\right),\;1\leq j\leq \left(n-m\right).$$
  If one or more matches occur during *m* segments, the following is obtained according to Equation (12) and the sum of *π* is 1.
  (18) $$\pi_{0}=1-e^{-\lambda \Delta t}\;,\;\pi_{j}=\frac{e^{-\lambda \Delta t}}{n-m}$$
  If no match occurs, then:
  (19) $$\pi_{0}=\frac{1-e^{-\lambda \Delta t}}{\left(m+1\right)}\;,\;\pi_{j}=\frac{e^{-\lambda \Delta t}}{\left(m+1\right)\left(n-m\right)}.$$
2. Recursion. For the hidden state sequence, *H*, and the observation sequence, *O*, the likelihood of *O* is estimated as:
  (20) $$P\left(O|H,\lambda \right)=\overset{n-m}{\underset{i=0}{\prod }}P\left(o_{i}|q_{i}\right)=\overset{n-m}{\underset{i=0}{\prod }}b_{i}\left(o_{i}\right).$$
  While introducing *π* and *A*, it follows:
  (21) $$P\left(H|\lambda \right)=\overset{n-m}{\underset{i=0}{\prod }}P\left(q_{i}|q_{i-1}\right)=\pi_{q_{0}}\overset{n-m}{\underset{i=1}{\prod }}a_{q_{i-1}q_{i}},$$
  where *q*_0_ is the initial state.
  Then the joint probability of *H* and *O* is:
  (22) $$P\left(O,H|\lambda \right)=P\left(O|H,\lambda \right)P\left(H|\lambda \right)=\overset{n-m}{\underset{i=0}{\prod }}b_{i}\left(o_{i}\right)\times \pi_{q_{0}}\overset{n-m}{\underset{i=1}{\prod }}a_{q_{i-1}q_{i}}.$$
  Therefore, the total probability of the observations can be calculated by summing up all possible hidden state sequences:
  (23) $$P\left(O|\lambda \right)=\underset{H}{\sum }P\left(O,H|\lambda \right)=\underset{H}{\sum }[\overset{n-m}{\underset{i=0}{\prod }}b_{i}\left(o_{i}\right)\cdot \pi_{q_{0}}\overset{n-m}{\underset{j=1}{\prod }}a_{q_{j-1}q_{j}}].$$
  For each given state *q_j_* at time *t*, the probability *α_t_*(*j*) is estimated as:
  (24) $$\alpha_{t}\left(j\right)=b_{j}\left(O_{t}\right)\overset{n-m}{\underset{i=0}{\sum }}\alpha_{t-1}\left(i\right)a_{ij},$$
  where 0 ≤ *j* ≤ (*n* − *m*) and 1 ≤ *t* ≤ *n*.
3. Termination. According to Equations (23) and (24), the probability of *O* is estimated as:
  (25) $$P\left(O|\lambda \right)=\overset{n-m}{\underset{j=0}{\sum }}\alpha_{t}\left(j\right).$$

Through the forward algorithm, the *P*(*O*|*λ*) of each observed state can be computed. In order to decide the popularity of each entry, the value of *O_i_* is set to 1 to record the probability of successful match. 

Large *P*(*O*|*λ*) means that the entry has high match probability. The number of popular flow entries selected in each flow table is denoted as *k*. Periodic ExTable update occurs in every Δ*T*. Here the popularity, *ω*, is decided based on the match frequency, *M*, and match probability, *P*(*O*|*λ*), as follows:

(26) $$\omega =\frac{M}{m}+P\left(O|\lambda \right).$$

After calculating the popularity, the flow entries of *k* largest popularity are moved to the ExTable. The number of flow entries in ExTable is *n_t_*∙*k* if there exist *n_t_* flow tables (*n_t_*∙*k* ≤ *n_p_*). If there exist several flow entries of the same value, the one of the longest remaining life time is selected. The proposed periodic entry selection scheme is depicted in Algorithm 2.

**Algorithm 2.** Selection operation of popular entry  **1: Begin**  **2:** create ExTable using the saved memory  **3:** set Δ*T*, *k*  **4:** *flowEntry.M*[*i*][*j*] = 0  **5:** *flowEntry.P*[*i*][*j*] = 0  **6:** *flowEntry.ω*[*i*][*j*] = 0  **7: while** 0 < Δ*t* < Δ*T*  **8:** **for**
*i* from 0 to *n_t_* −1  **9:**  **for**
*j* from 0 to *n_f_* −1 **10:**   input *O*, *λ* = (*π*,*A*,*B*)**11:**   initial *α*_0_(*j*) by Equation (17), *π* by Equations (18) and (19)**12:**   compute *α*_t_(*j*) by Equation (24)**13:**   calculate *flowEntry.P*[*i*][*j*] by Equation (25)**14:**   count *flowEntry.M*[*i*][*j*]**15:**   calculate *flowEntry.ω*[*i*][*j*] by Equation (26)**16:**  **end for****17:** **end for**
**18:**
**end while**
**19: if** (**Δ***t* == Δ*T*)**20:** **for***i* from 0 to *n_t_* − 1**21:**  sort *flowEntry. ω*[*i*][*j*] from large to small**22:** **for***j* from 0 to *k***23:**  select *flowEntry*[*i*][*j*]**24:** **end for****25:** **end for**
**26: end if**
**27:** update *flowEntry*[*i*][*j*] into ExTable
**28: end**


### 3.4. Flow Processing Time

**Figure 5:**
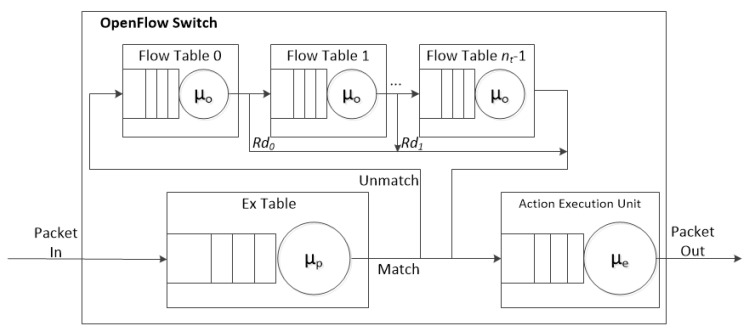
The queuing model of the proposed scheme.

The queuing model [19,37] of the proposed ExTable scheme is shown in Figure 5 where each of the nodes is considered as an M/M/1 queue. Table 7 is the list of variables used in the model.

According to Little’s Law [37], the flow processing time in the system can be calculated as *T* = *N*/*λ*, where *N* is the average number of flows in the system and *λ* is the arrival rate of the flows. For obtaining the flow processing time of the proposed scheme, *T_F_*, firstly the average number of flows in the system, *N_F_*, needs to be obtained. In the following formula *R_m_* is the match rate of the ExTable, and *ρ_p_, ρ_f_*, and *ρ_e_* are the utilization of ExTable, flow table, and AEU, respectively. Note that there exists a direct path from each flow table to AEU. The rate of sending packets directly to AEU from the flow tables are {*Rd*_0_, *Rd*_1_, …, *Rd_n_t__*_−2_}, where *n_t_* ≥ 2. In order to calculate *N_F_*, the average number of flows in ExTable, flow table 0, flow table 1, flow table (*n_t_*−1) and AEU, *N_p_*, *N_f_*_0_, *N_f_*_1_, *N_f_*_(*n_t_*−1)_, and *N_e_*, should be estimated. They are calculated as follows:

(27) $$N_{p}=\frac{\rho_{p}}{1-\rho_{p}}=\frac{\lambda n_{p}t_{f}}{1-\lambda n_{p}t_{f}},$$

(28) $$N_{f0}=\frac{\rho_{f0}}{1-\rho_{f0}}=\frac{\left(1-R_{m}\right)\lambda n_{f}t_{f}}{1-\left(1-R_{m}\right)\lambda n_{f}t_{f}},$$

(29) $$N_{f1}=\frac{\rho_{f1}}{1-\rho_{f1}}=\frac{\left(1-R_{m}-Rd_{0}\right)\lambda n_{f}t_{f}}{1-\left(1-R_{m}-Rd_{0}\right)\lambda n_{f}t_{f}},$$

(30) $$N_{f\left(n_{t}-1\right)}=\frac{\rho_{f\left(n_{t}-1\right)}}{1-\rho_{f\left(n_{t}-1\right)}}=\frac{\left[\left(1-R_{m}\right)-\sum_{i=0}^{n_{t}-2}Rd_{i}\right]\lambda n_{f}t_{f}}{1-\left[\left(1-R_{m}\right)-\sum_{i=0}^{n_{t}-2}Rd_{i}\right]\lambda n_{f}t_{f}},\;n_{t}\geq 2,$$

(31) $$N_{e}=\frac{\rho_{e}}{1-\rho_{e}}=\frac{\lambda t_{e}}{1-\lambda t_{e}}.$$

The average number of flows in the system, *N_F_*, is as follows:

(32) $$\begin{array}{ll} N_{F}=N_{p}+N_{f0}+ & N_{f1}+\cdots +N_{f\left(n_{t}-1\right)}+N_{e}\\ & =\lambda (\frac{n_{p}t_{f}}{1-\lambda n_{p}t_{f}}+\frac{\left(1-R_{m}\right)n_{f}t_{f}}{1-\left(1-R_{m}\right)\lambda n_{f}t_{f}}+\frac{\left(1-R_{m}-Rd_{0}\right)n_{f}t_{f}}{1-\left(1-R_{m}-Rd_{0}\right)\lambda n_{f}t_{f}}+\cdots \\ & +\frac{\left[\left(1-R_{m}\right)-\sum_{i=0}^{n_{t}-2}Rd_{i}\right]n_{f}t_{f}}{1-\left[\left(1-R_{m}\right)-\sum_{i=0}^{n_{t}-2}Rd_{i}\right]\lambda n_{f}t_{f}}+\frac{t_{e}}{1-\lambda t_{e}}). \end{array}$$

Note that there exist one ExTable, *n_t_* flow tables and one AEU. As a result, the flow processing time, *T_F_*, is obtained as follows:

(33) $$\begin{array}{ll} T_{F}=\frac{n_{p}t_{f}}{1-\lambda n_{p}t_{f}}+ & \frac{\left(1-R_{m}\right)n_{f}t_{f}}{1-\left(1-R_{m}\right)\lambda n_{f}t_{f}}+\frac{\left(1-R_{m}-Rd_{0}\right)n_{f}t_{f}}{1-\left(1-R_{m}-Rd_{0}\right)\lambda n_{f}t_{f}}+\cdots \\ & +\frac{\left[\left(1-R_{m}\right)-\sum_{i=0}^{n_{t}-2}Rd_{i}\right]n_{f}t_{f}}{1-\left[\left(1-R_{m}\right)-\sum_{i=0}^{n_{t}-2}Rd_{i}\right]\lambda n_{f}t_{f}}+\frac{t_{e}}{1-\lambda t_{e}}. \end{array}$$

The queueing model of the flow processing time for ExTable is used later in computer simulation. The proposed scheme is simulated and compared with the existing schemes in the following section.

## 4. Performance Evaluation

In this section computer simulation is conducted to evaluate the TCAM compression ratio, prediction accuracy, and match rate of the proposed approach.

### 4.1. Simulation Environment

The simulation is conducted on Intel Core i5 process, 3.2 GHz PC with 8GB RAM, and Matlab R2014a. The flows used in the simulation are generated following exponential distribution with *λ* = 1. A virtual SDN environment is built with Floodlight controller, Open vSwitch, and end nodes emulated by Mininet. Here the Floodlight controller is linked to an Open vSwitch, and two end nodes are connected to the switch. The performance of the proposed scheme is compared with the original MFT approach and existing schemes [20,21]. 

For testing the proposed entry aggregation scheme, eight different numbers of flow entries are generated randomly. The number of entries is varied from 100 to 800. For obtaining the flow processing time for MFT and ExTable, the queueing models of them are used in the simulation. The number of flow entries in a flow table is set to 20, while the size of ExTable is *n_t_*∙*k*. The service rate of the ExTable, flow table, and AEU are set to be 1.67, 1.67, and 10 [19,21], respectively. Table 8 and Table 9 list the parameter values and the factors used in the simulation, respectively.

The match rate and prediction accuracy of the proposed scheme are examined with various values of the operation parameters, and the flow processing time is compared with the existing schemes. Note that small processing time implies higher match rate and prediction accuracy.

The simulation is run 1000 times to achieve dependable result. The accuracy of the proposed HMM-based prediction is then calculated as:

(34) $$Accuracy=\frac{N\_Success}{N\_Experiment}\times 100\%.$$

Here, *N_Success* indicates the number of selected flow entries actually having the largest number of matches, and *N_ Experiment* is the whole number of simulations. The match rate of the proposed ExTable scheme is obtained as follows:

(35) $$Match\;rate=\frac{N\_Match}{N\_Flows}\times 100\%.$$

Here, *N_Match* is the number of incoming flows matching the ExTable, while *N_Flows* is the total number of incoming flows.

### 4.2. Simulation Results

**Figure 6:**
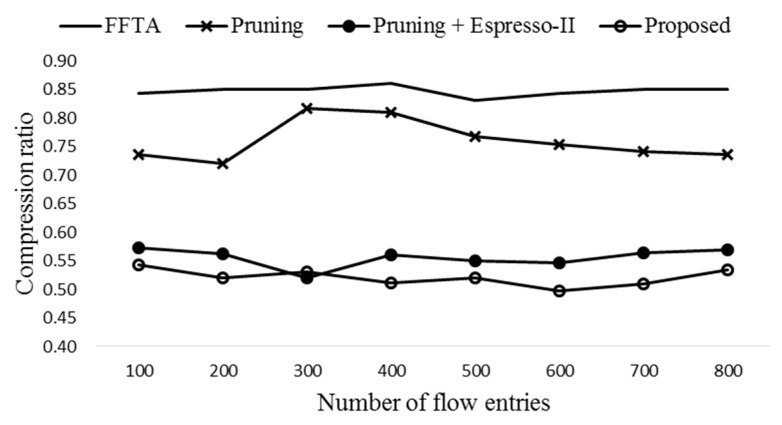
The compression ratios of four different schemes.

Figure 6 shows the average compression ratios with eight different numbers of flow entries. In order to achieve dependable result, the simulation is run 1000 times for each number of flow entries generated randomly. Here compression ratio is the number of entries after reduction to that of original entries [8,32]. Therefore, lower compression ratio means more entries are aggregated. The pruning alone reduces the table size by almost 25%. The proposed scheme (pruning + QM) shows the best compression ratio among the four schemes compared.

**Figure 7:**
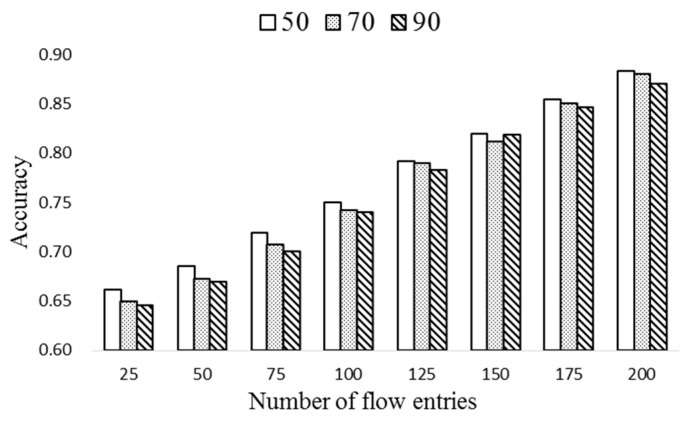
The prediction accuracy vs size of flow table.

Figure 7 shows the prediction accuracy of the proposed HMM-based scheme with different sizes of flow table. The number of flows is 500 and Δ*T* (ExTable update period) is set to 20. In order to see the effect of *T_H_*, it is set to 50, 70, and 90. Observe that, as the number of entries of the flow table increases, the prediction accuracy increases. Also, the accuracy slightly decreases as *T_H_* increases because many obsolete flow entries may remain in the flow tables as time goes by. 

**Figure 8:**
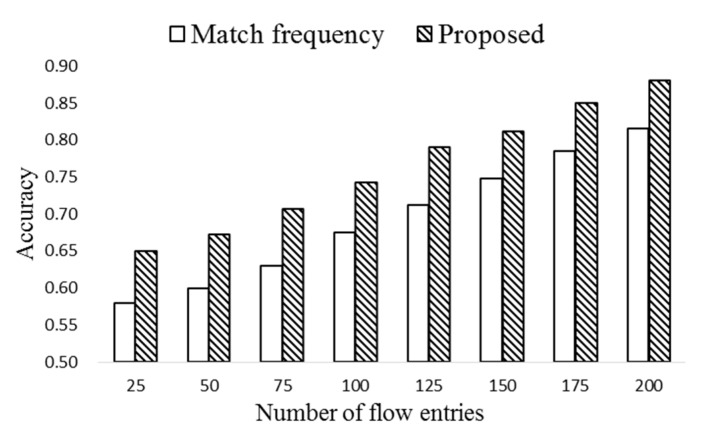
The comparison of prediction accuracies vs size of flow table.

Figure 8 compares the prediction accuracy of the proposed HMM-based scheme with the scheme based on only match frequency. Here the number of flows is 500 and (Δ*T*,*T_H_*) are set to (20,70). Notice from the figure that the proposed scheme shows consistently higher accuracy, while the accuracy of both the schemes increases as the number of flow entries grows. Note that high match frequency may not be the only indicator of the popularity of an entry. Even though an entry has low match frequency, it could be a popular one if its match probability is high. 

**Figure 9:**
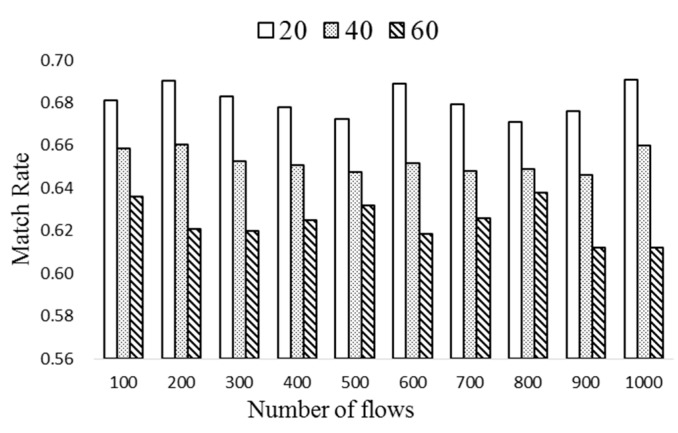
The match rates of ExTable with different Δ*T*.

In Figure 9, the match rate of popular flow entries is obtained with different Δ*T* of 20, 40, and 60. According to the result of Figure 6, the space saving due to the compression is about 45%. Since *n_t_*∙*k* ≤ 0.45∙20*n_t_*, *n_t_* and *k* are set to 5 and 9, respectively. Observe from the figure that the match rate decreases as Δ*T* increases, which demonstrates that ExTable needs to be updated as quickly as possible. The proposed scheme achieves nearly 68% match rate when Δ*T* is 20. As Δ*T* becomes large, some entries may not be popular any more. There exists a trade-off between the match rate and update cost. 

**Figure 10:**
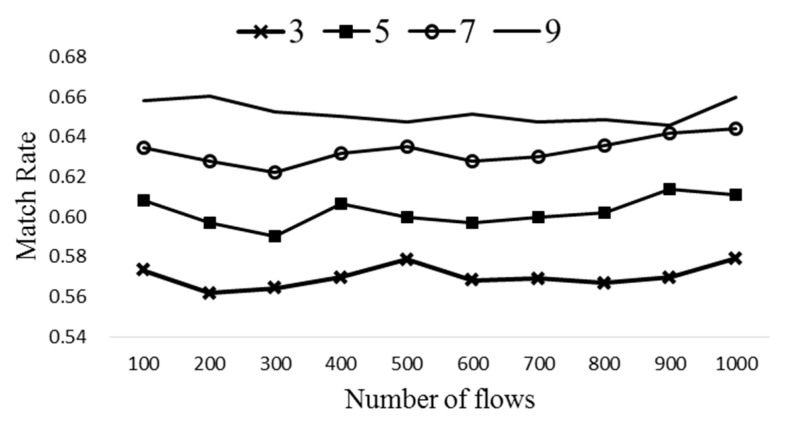
The match rates with different *k*.

Figure 10 evaluates the match rate of ExTable with different number of popular entries per flow table (*k*). Note that *k* can be varied from 1 to 9 each allowing different compression ratio. Here *n_t_* is set to 5 and Δ*T* to 40, while *k* is varied from 3 to 9. Notice from Figure 10 that the match rate increases as *k* grows as expected. A proper number of popular entries needs to be selected which allows high match rate while requiring reasonable operation cost. 

**Figure 11:**
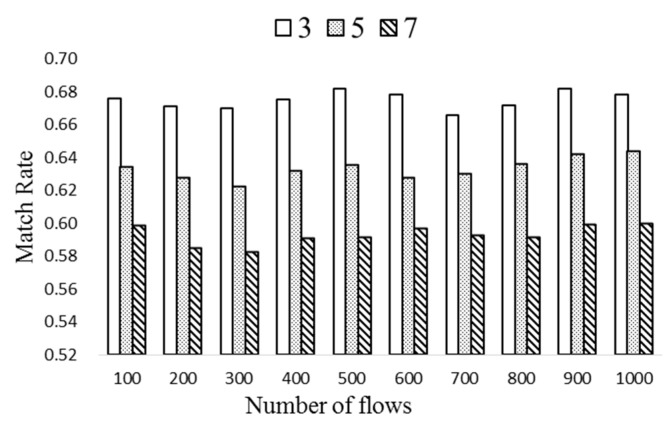
The match rates with different *n_t_*.

In Figure 11, the match rate is obtained with different number of flow tables, *n_t_*, of 3, 5, and 7. Here Δ*T* is 40 and *k* is 7. It is clear that the match rate of ExTable decreases as *n_t_* grows since the number of total flow entries becomes larger. Therefore, *k* needs to be set properly considering various design parameters. 

**Figure 12:**
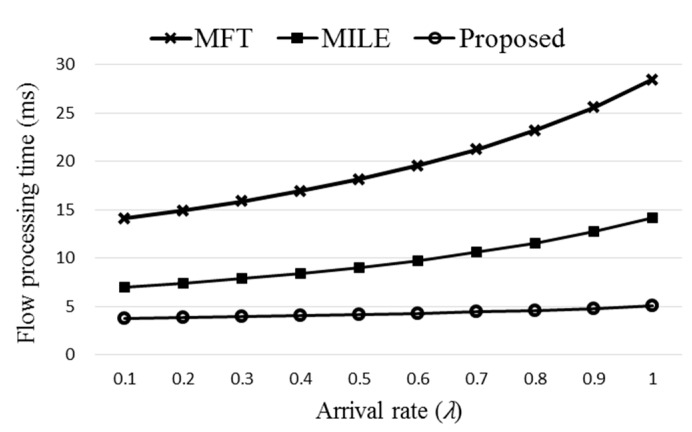
The comparison of flow processing times with varied arrival rate.

**Figure 13:**
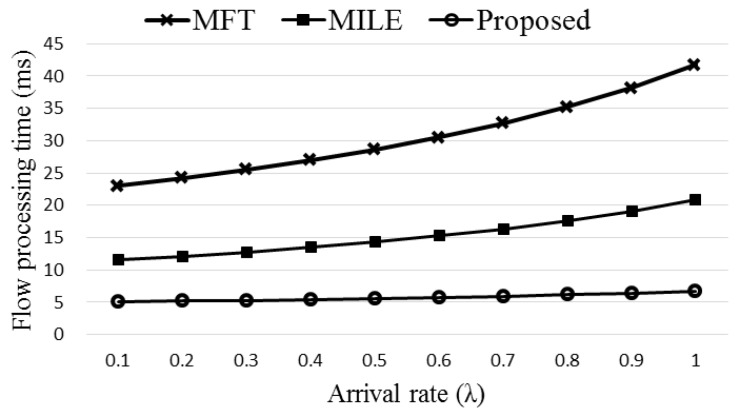
The comparison of flow processing times with varied arrival rate.

Figure 12 compares the flow processing time of the three schemes, original MFT, MILE, and the proposed Agg-ExTable. Here (Δ*T*,*R_m_*,*n_p_*,*n_f_*,*k*,*n_t_*) of the proposed scheme are set to (20,0.68,35,20,7,5) using the data obtained from the simulation. The rate of using the direct path, *Rd_n_*, is decided randomly between 0 and 0.1. Observe that the proposed Agg-ExTable scheme achieves much smaller flow processing time than the other schemes as the ExTable provides an express forwarding path for a large portion of incoming flows. The difference gets more significant as the flow arrival rate increases. Note that high arrival rate means high network load, and the flow processing time of all the schemes grows as the network load increases. Another important merit of the proposed scheme is that the processing time is almost constant regardless of the load unlike the other schemes. Figure 13 shows the flow processing times with different settings of (40,0.6,35,20,5,7). The proposed scheme consistently outperforms the other schemes. 

## 5. Conclusions

In this paper a novel flow management scheme has been proposed for MFTs of SDN switches. In the proposed Agg-ExTable scheme, the flow entries in the MFT are periodically aggregated by applying the pruning and Quine–Mccluskey algorithm. Utilizing the memory space saved by the aggregation, ExTable is constructed, keeping popular flow entries and allowing early match with the incoming flows. The proposed scheme is able to save about 45% TCAM space of MFT and efficiently decrease the flow processing time through the express forwarding path provided by the front-end ExTable. Popular flow entries are selected from the flow tables using the HMM, where popularity is decided based on the match frequency and match probability. Computer simulation revealed that the proposed scheme significantly outperforms the existing schemes in terms of flow processing time.

In the future, we plan to investigate the approach further reducing the memory space used for ExTable. Various parameters are involved in the proposed scheme. A formal model will be developed with which proper parameter values can be decided for the given condition. In addition, the match rate of the ExTable will be further improved with more sophisticated techniques such as machine learning and fuzzy theory in the selection of popular entries. The proposed approach will also be tested with a real test bed for various operational conditions and SDN environments.

## Figures and Tables

**Table 1:** An example of flow table with TCAM.

No.	Entry/Mask								Action
*E*_1_	1	0	X	1	1	X	X	X	Forward to 1
	1	1	0	1	1	0	0	0	
*E*_2_	0	X	0	0	X	X	X	0	Forward to 2
	1	0	1	1	0	0	0	1	
*E*_3_	1	1	X	X	1	1	X	X	Forward to 5
	1	1	0	0	1	1	0	0	
*E*_4_	1	X	X	X	0	X	X	X	Forward to j
	1	0	0	0	1	0	0	0	
*E*_5_	X	X	X	1	1	X	X	X	Forward to k
	0	0	0	1	1	0	0	0	

**Table 2:** An example of mask extension.

**Before**				**Mask extension** 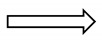	**After**			
**No.**	**Prefix**	**Mask**	**Action**		**No.**	**Prefix**	**Mask**	**Action**
*E*_1_	1100	1111	Forward to 1		*E*_1&2_	1100	1101	Forward to 1
*E*_2_	1110	1111	Forward to 1		*E*_3_	1000	1000	Forward to 2
*E*_3_	1000	1000	Forward to 2		*E*_4&5_	1101	1011	Forward to 3
*E*_4_	1101	1111	Forward to 3					
*E*_5_	1001	1111	Forward to 3					

**Table 3:** An example table.

No.	A	B	C	D	Action
*E*_0_	0	1	0	1	Forward to 1
*E*_1_	0	0	0	0	Forward to 1
*E*_2_	0	0	1	0	Forward to 2
*E*_3_	0	1	1	1	Forward to 1
*E*_4_	0	1	0	0	Forward to 1
*E*_5_	1	1	1	1	Forward to 1
*E*_6_	1	0	1	0	Forward to 1
*E*_7_	1	0	0	0	Forward to 1
*E*_8_	1	0	1	1	Forward to 1
*E*_9_	1	1	0	1	Forward to 1
*E*_10_	1	0	0	1	Forward to 3

**Table 4:** Minterm table.

Stage I					Stage II (Size 2 Implicants)				
No. of 1s	Ʃ(*E_i_*)		ABCD	*P_i_*	No. of 1s	Ʃ(*E_i_*)		ABCD	*P_i_*
0	1		0000	/	0	1, 4		0-00	*P*_7_
						1, 7		-000	*P*_6_
1	4		0100	/	1	4, 0		010-	*P*_5_
	7		1000	/		7, 6		10-0	*P*_4_
2	0		0101	/	2	0, 3		01-1	/
	6		1010	/		0, 9		-101	/
3	3		0111	/		6, 8		101-	*P*_3_
	8		1011	/	3	3, 5		-111	/
	9		1101	/		8, 5		1-11	*P*_2_
4	5		1111	/		9, 5		11-1	/
Stage III (Size 4 Implicants)									
No. of 1s		Ʃ(*E_i_*)			ABCD		*P_i_*		
2		0, 3, 9, 5			-1-1		*P*_1_		

**Table 5:** The essential prime implicant table.

***P_i_***	***E_i_***								
	0	1	3	4	5	6	7	8	9
*P*_1_	*		*		*				*
*P*_2_					*			*	
*P*_3_						*		*	
*P*_4_						*	*		
*P*_5_	*			*					
*P*_6_		*					*		
*P*_7_		*		*					

**Table 6:** The final table.

*E_i_*	A	B	C	D	Action
*E*_*p*1_	-	1	-	1	Forward to 1
*E*_*p*2_	1	-	1	1	Forward to 1
*E*_*p*3_	1	0	-	0	Forward to 1
*E*_*p*4_	0	1	0	-	Forward to 1
*E*_2_	0	0	1	0	Forward to 2
*E*_10_	1	0	0	1	Forward to 3

**Table 7:** The variables used in the queueing model.

Variable	Description
*n_t_*	Number of original flow tables
*λ*	Arrival rate of the flows
*n_p_*	Number of flow entries in ExTable
*t_f_*	Matching time with a flow entry
*n_f_*	Number of flow entries in a flow table
*t_e_*	Execution time of action with a packet
*Rd_n_*	Rate of using direct path in each flow table
*R_p_*	Match rate of the ExTable

**Table 8:** The parameter setting of the simulation.

Parameter	Value
*n_p_*	*n_t_*∙*k*
*n_f_*	20
*μ* *_p_*	1.67
*μ_f_*	1.67
*μ_e_*	10

**Table 9:** The factors in the simulation.

Variable	Description
*C*	Compression ratio
*k*	Number of popular flow entries per table
*n_t_*	Number of flow tables
*λ*	Flow arrival rate
*t_h_*	Hard timeout value
*R_d_*	Rate of using direct path in each flow table
Δ*T*	ExTable update period
